# SARS-CoV-2 Variants: A Synopsis of In Vitro Efficacy Data of Convalescent Plasma, Currently Marketed Vaccines, and Monoclonal Antibodies

**DOI:** 10.3390/v13071211

**Published:** 2021-06-23

**Authors:** Daniele Focosi, Marco Tuccori, Andreina Baj, Fabrizio Maggi

**Affiliations:** 1 North-Western Tuscany Blood Bank, Pisa University Hospital, Via Paraisa 2, 56124 Pisa, Italy; 2 Division of Pharmacovigilance, Pisa University Hospital, Via Paradisa 2, 56124 Pisa, Italy; m.tuccori@ao-pisa.toscana.it; 3 Department of Medicine and Surgery, University of Insubria, 21100 Varese, Italy; andreina.baj@uninsubria.it (A.B.); fabrizio.maggi@uninsubria.it (F.M.)

**Keywords:** COVID-19, SARS-CoV-2, convalescent plasma, neutralizing antibody, immune escape, mutations, LyCoV016, etesevimab, REGN10987, imdevimab

## Abstract

We summarize here in vitro evidences of efficacy for convalescent plasma, currently approved vaccines and monoclonal antibodies against SARS-CoV-2 variants of concern (VOC: B.1.1.7, B.1.351, P.1, and B.1.617.2), variants of interest (VOI: B.1.427/B.1.429, P.2, B.1.525, P.3, B.1.526, and B.1.671.1), and other strains (B.1.1.298 and B.1.258delta). While waiting from real world clinical efficacy, these data provide guidance for the treating physician.

The ongoing SARS-CoV-2 pandemic has entered a new dimension thanks to availability of different vaccines and neutralizing antibody-based therapeutics (from convalescent plasma to monoclonal antibodies). Nevertheless, emerging of SARS-CoV-2 variants of concern (VOC) and variants of interest (VOI) [1] has diversified the landscape, jeopardizing the efforts to contain it. Media-informed patients are questioning physicians about the relative efficacy of different vaccines and treatments against different variants.

The majority of registration trials for currently approved monoclonal antibodies and vaccines were run either before the variants emerged, or enrolled patients in countries where such variants were not circulating at that time. While waiting for post-marketing clinical efficacy data (i.e., protection from symptomatic COVID19), many investigators have tested the neutralizing efficacy of monoclonal antibodies, convalescent plasma from previous waves or vaccinee sera in vitro to accelerate availability of surrogate endpoints. Different nonviral (e.g., synthetic RBD) and viral constructs (ranging from isogenic strain, pseudovirus harboring the full mutation set, or authentic variant virus) have been employed to test the efficacy of therapeutics in neutralization assays [2].

On 15 May 2021, we mined PubMed and preprint servers (medrXiv and biorXiv) for in vitro data detailing the efficacy of different anti-Spike vaccines and monoclonal antibodies against different variants compared to wild-type SARS-CoV-2. Table 1 reports the result of our search and analysis. We decided to simplify interpretation of results using a semiquantitative scale according to the number of fold decrease in neutralization efficacy. We also tabulated for each variant the estimated reinfection rates, and the proven reinfection cases (strains from each episode sequenced). Each variant was reported using both the official (PANGOLIN and NextStrain) and the local (VUI/VOC/VOI) naming systems, and colloquial terms (e.g., “UK variant”) in order to provide comprehensive association. The main, alarming finding is the lack of efficacy of single-agent bamlanivimab against most E484K-carrying variants. Accordingly, the FDA has recently withdrawn its emergency use authorization as a single agent, leaving the authorization only for usage in combination with etesevimab. Nevertheless, Q493R mutation, causing resistance to both mAbs, has been recently reported by our group [3]. High-frequency Spike mutations R346K/S, N439K, G446V, L455F, V483F/A, E484Q/V/A/G/D, F486L, F490L/V/S, Q493R, and S494P/L might compromise some of mAbs in clinical trials [4].

We could not find any in vitro evidence of efficacy against SARS-CoV-2 variants for several widely used vaccines (e.g., Sinopharm’s BBIBP-CorV), stressing the need for more studies. 

Meta-analysis of 56 vaccine studies, including 2483 individuals and 8590 neutralization tests, showed that, compared with lineage B, there was a 1.5-fold reduction in neutralization against the B.1.1.7, 8.7-fold reduction against B.1.351 and 5.0-fold reduction against P.1. The estimated neutralization reductions for B.1.351 compared to lineage B were 240.2-fold reduction for non-replicating vector platform, 4.6-fold reduction for RNA platform, and 1.6-fold reduction for protein subunit platform. The neutralizing antibodies induced by administration of inactivated vaccines and mRNA vaccines against lineage P.1 were also remarkably reduced by an average of 5.9-fold and 1.5-fold [5]. Efficacy of convalescent plasma from previous waves is also generally lowered: convalescent plasma from a donor affected during the early 2020 protected against SARS-CoV-2 WA-1 wild-type strain but was insufficient to protect against challenge with B.1.1.7 and B.1.351 in a mouse model [6].

The relevance of these in vitro data in real life remains unclear. Khoury et al, assuming that the neutralization level required for 50% protection against detectable SARS-CoV-2 infection was 20.2% of the mean convalescent level and that the neutralization level for 50% protection from severe infection was 3% of the mean convalescent level, reported that the decay of the neutralization titer over the first 250 days after immunization predicts a significant loss in protection from SARS-CoV-2 infection, although protection from severe disease should be largely retained. Neutralization titers against some SARS-CoV-2 VOC are reduced compared with the vaccine strain [7]. A case-control study showed that, compared with unvaccinated individuals, BNT162b2 vaccinees with documented SARS-CoV-2 infection at least a week after the second dose were disproportionally infected with B.1.351 (odds ratio of 8:1), while those infected between 2 weeks after the first dose and 1 week after the second dose, were disproportionally infected by B.1.1.7 (odds ratio of 26:10), suggesting reduced vaccine efficacy (VE) against both VOCs under different dosage/timing conditions. Nevertheless, the B.1.351 incidence in Israel to-date remains low and VE remains high against B.1.1.7, among those fully vaccinated. These results overall suggest that vaccine breakthrough infection is more frequent with both VOCs, yet a combination of mass-vaccination with two doses coupled with non-pharmaceutical interventions control and contain their spread [8]. With mRNA-1273, binding of vaccine-elicited anti-RBD antibodies is more broadly distributed across epitopes than for infection-elicited anti-RBD antibodies [9]: accordingly, greater IgG2, IgG3, and IgG4 responses and higher ratios of (IgG1 + IgG3)/(IgG2 + IgG4) were seen in subjects vaccinated with either BNT162b2 or mRNA-1273 than in convalescents [10]. This greater binding breadth means single RBD mutations have less impact on neutralization by vaccine sera than convalescent sera. Another striking feature is that for BNT162b2 post-first dose vaccination infection did not increase IgG titres, so that individuals infected post-dose one should receive the second [11]. CoronaVac for which no in vitro efficacy data are available, was 42% effective in the real-world setting of extensive P.1 transmission, but significant protection was not observed until completion of the two-dose regimen [12]. A metaanalysis by Shapiro et al found that, on average, the VE against any disease with infection was 85% after a full course of vaccination. The VE against severe disease, hospitalization or death averages close to 100%. The average VE against infection, regardless of symptoms, was 84%. The average VE against [13] B.1.1.28 (P1) and B.1.351 were 86% (95% CI: 65–84%), 61% (95% CI: 43–73%) and 56% (95% CI: 29–73%), respectively [14].

Additionally, a single injection of mRNA-1273 or BNT162b2 has been shown enough to induce novel antibody specificities that protect against the B.1.351 VOC [15]: a similar phenomenon has been reported after 2 BNT162b2 doses against B.1.1.7 [16]. Neutralizing antibody titers increased in previously infected BNT162b2 vaccinees relative to uninfected vaccinees against every variant tested: 5.2-fold against B.1.1.7, 6.5-fold against B.1.351, 4.3-fold against P.1, and 3.4-fold against original SARS-CoV-2 [17]. Similarly, a single dose of either BNT162b2 or AZD1222 vaccines in convalescents raised the titre of antibodies against the SARS-CoV-2 vaccine strain (B.1) and three major VOCs (B.1.1.7, B.1351 and P.1). A single dose to convalescents is nowadays a well-accepted approach that saves money and side effects [18].

Apart from efficacy, many topics remain under investigation for vaccines:

- vaccine-elicited T-cell immunity: while neutralizing antibodies are just one arm of the adaptive immune response to vaccines, very few data are available for protection from T-cell immunity, which would be especially relevant in the ones who do not mount antibody responses. Gallagher et al found detectable but diminished T-cell responses to Spike variants (B.1.1.7, B.1.351, and B.1.1.248) among BNT162b2 or mRNA-1273 vaccinated donors [19]. BNT162b2 or mRNA-1273-elicited spike-specific T cells responded similarly to stimulation by Spike epitopes from the ancestral, B.1.1.7 and B.1.351 variant strains, both in terms of cell numbers and phenotypes. In infection-naive individuals, the second dose boosted the quantity but not quality of the T cell response, while in convalescents the second dose helped neither. Spike-specific T cells from convalescent vaccinees differed strikingly from those of infection-naive vaccinees, with phenotypic features suggesting superior long-term persistence and ability to home to the respiratory tract including the nasopharynx [20].
- duration of protection: according to a mathematical model by Luo et al, after mRNA-1273 vaccination, pseudovirus neutralization test against B.1.351 is expected to fall below the lower limit of detection of 20 geometric mean titers on day 100; variant P.1 on day 202, variant B.1.429 on day 258; and variant B.1.1.7 on day 309 [21]. Real-world data instead suggested that binding and functional antibodies against B.1.1.7, B.1.351, P.1, B.1.429, and B.1.526 variants persisted in most subjects, albeit at low levels, for 6 months after the primary series of mRNA-1273 [22].
- postponing second doses has been widely implemented in order to optimize vaccine delivery under manufacturing bottlenecks. In nonconvalescent elderlies higher than age 80 who received the second dose of BNT162b2 after 12 weeks instead of 3, the peak antibody response was 3.5-fold higher, but cellular immune responses were 3.6-fold lower [23].
- heterologous boosting: heterologous immunization strategy combining inactivated and mRNA vaccines can generate robust vaccine responses and therefore provide a rational and effective vaccination regimen [24]. ChAdOx/BNT162b2 booster vaccination was largely comparable to homologous BNT162b2/BNT162b2 vaccination and overall well-tolerated. No major differences were observed in the frequency or severity of local reactions after either of the vaccinations. In contrast, notable differences between the regimens were observed for systemic reactions, which were most frequent after prime immunization with ChAdOx (86%) and less frequent after homologous BNT162b2/BNT162b2 (65%), or heterologous ChAdOx/BNT162b2 boosters (48%) [25]. Neutralizing activity against the prevalent strain B.1.1.7 was 3.9-fold higher than in individuals receiving homologous BNT162b2 vaccination, only 2-fold reduced for variant of concern B.1.351, and similar for variant B.1.617 [26]. Whilst both ChAdOx and BNT162b2 boosted prime-induced immunity, BNT induced significantly higher frequencies of Spike-specific CD4 and CD8 T cells and, in particular, high titers of neutralizing antibodies against the B.1.1.7, B.1.351 and the P.1 VOCs [27].

**Table 1:** Synopsis of efficacy of vaccines and monoclonal antibodies against current SARS-CoV-2 VOCs, VOIs and emerging strains.

			Variants of Concern (VOC)				Variants of Interest (VOI)						Other	
**Therapeutic\Variant**		PANGOLIN name	B.1.1.7	B.1.351	P.1	B.1.617.2	P.2	B.1.427 B.1.429	P.3	B.1.525	B.1.526 with E484K or S477N	B.1.617.1	B.1.258D	B.1.1.298
		NextStrain name	20I/S:501Y.V1	20H/S:501Y.V2	20J/S:501Y.V3	21A/S:478K	20B/S.484K	20C/S.452R	20B/S:265C	20A/S484K	20C/S.484K	21A/S:154K	-	-
		PHE name	VOC-20DEC-01	VOC-20DEC-02	VOC-21JAN-02	VUI-21APR02	VUI-21JAN-01	-	-	VUI-21FEB-03	-	-	-	-
		WHO name	alpha	beta	gamma	delta	zeta	epsilon	theta	eta	iota	kappa	-	-
		GISAID name	GRY (formerly GR/501Y.V1)	GH/501Y.V2	GR/501Y.V3	G/452R.V3	GR	GH/452R.V1	GR	G/484K.V3	GH	G/452R.V3	-	-
		local name	VUI/VOC 202012/01, UK variant	501Y.V2 VOC 202012/02	B.1.1.28.1 B.1.1.248 VOC 202101/02	Indian variant	B.1.1.28.2 B.1.1.28(E484K)	CAL.20C/L452R, West Coast variant	-	Nigerian variant	-	Indian variant	-	Cluster V
**country of first detection**			South-East England, UK	South Africa	Amazonas, Brazil	India	Rio de Janeiro, Brazil	Southern California, USA	Brazil	Nigeria	New York, USA	India	Czech Republic, Slovakia	Denmark
**convalescent plasma (sera) from previous waves**			↓ [28,29,30,31,32,33,34,35]	↓↓↓ [29,31,33,34,36,37,38,39,40,41]	↓↓↓ [42]	= [43] ↓[44,45,46] ↓↓ [41]	= [47] ↓↓↓ [48] (hamsters)	↓↓ [49]	?	?	↓↓ [50,51]	?	↓ [52]	?
**hyperimmune serum (polyvalent immunoglobulins)**			= [53]	↓↓↓ [53]	↓↓↓ [53]	?	?	= [53]	?	?	?	?	?	?
**estimated reinfection rate**			0.7% [54]	?	6.4% [55]	?	?	?	?	?	?	?	?	?
**proven reinfection**			1 case [56]	1 case [57]	1 case [58]	?	2 cases [59,60]	?	?	?	?	?	?	?
**mAbs-**	Eli Lilly (AbCellera/Junshi)	etesevimab (LyCoV016, CB6 or JS016/LY3832479)	↓↓↓ [29,61]	= [29]	↓↓↓ [42,61]	?	?	↓↓ [49]	?	?	= [51]	?	?	?
		bamlanivimab (LY-CoV555/LY3819253)	↓↓↓ [29,37,62,63]	= [29,63]	↓↓↓ [37,42,62]	↓↓↓ [44,45,46,64]	↓↓↓ [63]	↓↓↓ [49,65]	?	?	↓↓↓ [50,51]	?	?	?
		LY-CoV1404	= [66]	= [66]	= [66]	?	?	= [66]	?	?	= [66]	?	?	?
	Regeneron/Roche REGN-CoV2	imdevimab (REGN10987)	= [29]	= [29]	= [42,61]	?	?	= [49]	?	?	= [50,51]	?	↓↓↓ [52]	?
		casirivimab (REGN10933)	↓↓↓ [61]	↓↓ [29]	↓↓↓ [37,42,61]	?	?	= [49]	?	?	↓↓↓ [50,51]	?	?	?
	Celltrion	regdanvimab (CT-P59)	↓↓ [67]	?	?	?	?	↓↓ [49]	?	?	?	?	?	?
	A-straZeneca AZD7442 long-acting antibody (LAAB)	AZD8895/COV2-2196	↓↓↓ [29]	↓↓↓ [29]	= [42]	?	?	?	?	?	?	?	?	?
		AZD1061/COV2-2130	= [29]	= [29]	= [42]	?	?	?	?	?	?	?	?	?
	BMS	C135	= [29]	= [29]	?	?	?	?	?	?	?	?	?	?
		C144	?	?	?	?	?	?	?	?	?	?	?	?
	Vir Biotechnology	VIR-7831/GSK-4182136 (sotrovimab) and VIR-7832/GSK-4182137 (both derived from S309)	= [29,68]	= [29,68]	= [42,68]	?	?	?	?	?	?	?	?	?
**vaccine**	BioNtech/Pfizer	BNT162b2/tozinameran (Comirnaty^®^)	= [69] ↓ [15,29,31,33,35,37,63,70,71,72,73,74,75] RBD ↓↓↓ [76,77,78] COVID19 75% [79]	=/↓ [29,31,32,33,35,63,70,71,72,73,74,75,80,81,82] RBD [78] COVID19 90% [79,83]-93% [84]	↓↓ [35,42,75,76,85]	↓↓↓ [44,45,46,86,87] COVID19 88% [84]	↓ [63]	=/↓↓ [49,75] RBD [78]	?	↓ [88]	↓↓ [50,51]	?	?	= [75]
	Moderna	mRNA-1273	↓ [15,29,38,74,75,89] ↓ NHP COVID19 [90]	= [29,30,89] ↓ [74,75,91,92]	=/↓ [42,75]	↓↓ [44,87]	?	=/↓↓ [49,75]	?	?	↓↓ [50,51]	?	?	= [75]
	AstraZeneca	AZD1222/ChAdOx1 (Vaxzevria^®^, Covishield^®^)	↓↓↓ [93] = hamster COVID19 [94] COVID19 22%	? COVID19 90% [83]-66% [84] = hamster COVID19 [94]	↓↓ [85]	↓ [45,95] COVID19 % [84]	?	?	?	?	?	?	?	?
	Gamaleya	Sputnik V/Gam-Covid-Vac	↓↓↓ [96]	= [96]	?	?	?	?	?	?	?	?	?	?
	Novovax	NVX-CoV2373 (Covovax^®^)	? COVID19: 51% [97]	↓ [92]	?	?	?	?	?	?	?	?	?	?
	Bharat Biotech	BBV152/Covaxin	?	= [98] ↓ [41]	?	= [43] ↓ [41]	↓ [47]	?	?	?	?	?	?	?
	SinoVac	CoronaVac	↓↓↓ [99]	= [99]	↓↓ [99]	?	?	= [99]	?	?	↓↓ [99]	?	?	?
	J&J/Janssen	JNJ-78436735/Ad26.COV2.S	?	↓↓↓ [100]	?	?	?	?	?	?	?	?	?	?

VOC: variants of concern; VOI: variant of interest; NHP: nonhuman primates. = and arrows indicate fold-reductions in neutralizing activity compared to wild-type D614G strain. =: no reduction; ↓: 1-3 fold reduction; ↓↓: 3-5 fold reduction; ↓↓↓: > 5 fold reduction; ?: data not available). COVID19 refers to vaccine efficacy against symptomatic diseases in humans (if not otherwise indicated) or in animal models (specified). RBD: ACE2-RBD competition assay.

While the in vitro findings summarized here wait for confirmatory clinical evidences, in the meanwhile they could orient therapeutic and preventive strategies.

## Data Availability

The data presented in this study are openly available in PubMed.

## Abbreviations

nAb	neutralizing antibodies
CCP	COVID19 convalescent plasma
RBD	receptor-binding domain
RBM	receptor-binding motif
